# Right lower transverse incision versus vertical transumbilical incision for laparoscopic specimen extraction in patients with left-sided colorectal cancer: a comparative study of two mini-laparotomy techniques

**DOI:** 10.1186/s12957-016-1030-2

**Published:** 2016-10-26

**Authors:** Jin Yong Shin

**Affiliations:** Department of Surgery, Inje University College of Medicine, Inje University Haeundae Paik Hospital, 875 Haeundae-ro, Haeundae-gu, Busan, 612-896 Republic of Korea

**Keywords:** Specimen extraction, Laparoscopic surgery, Colorectal cancer, Short-term outcomes

## Abstract

**Background:**

The aim of this study was to compare the short-term outcomes of a right lower transverse incision with a vertical transumbilical incision for laparoscopic specimen extraction in patients with left-sided colorectal cancer.

**Methods:**

One hundred eighty-three patients who underwent laparoscopic resection for rectosigmoid colon or upper rectal cancer were included. Propensity score matching was performed to reduce bias caused by differences between the right lower transverse incision (RLT group) and vertical transumbilical incision (VTU group).

**Results:**

After matching, 57 patients in the RLT group and 57 patients in the VTU group were found to be equivalent regarding baseline clinicopathological characteristics. Median follow-up time was 31 months. The RLT group showed comparable results to those of the VTU group in terms of perioperative outcomes, postoperative course, and postoperative complications. However, the proportion of patients requiring an additional incision for diverting stoma was significantly lower in the RLT group (*p* = 0.003).

**Conclusions:**

A right lower transverse incision appears to be as effective as a vertical transumbilical incision regarding short-term outcomes after laparoscopic surgery for left-sided colorectal cancer and may be a preferred extraction site because of its lowered risk of additional mini-laparotomy for diverting stoma.

## Background

The performance of laparoscopic surgery for the treatment of colon cancer has become widespread because of its superior short-term surgical outcomes and comparable long-term surgical outcomes [1–3]. Laparoscopic surgery for mid or lower rectal cancer with the probable need for neoadjuvant therapy is still considered challenging [4], while laparoscopic surgery for rectosigmoid or upper rectal cancer has been regarded more straightforward based on similarities in surgical and oncologic outcomes between these cancers and sigmoid colon cancer [5–8]. Following laparoscopic resection for colorectal cancer, mini-laparotomy is often necessary to extract the resected colon or rectum and perform anastomosis. The preferred specimen extraction site after laparoscopic colorectal resection is reported to be either the low transverse or midline area [9–15]. However, laparoscopic extraction sites may cause problems like incisional hernia, wound infection, and tumor implantation at the wound sites [11, 13, 16].

In patients undergoing laparoscopic surgery for left-sided colorectal cancer, a low transverse or vertical incision is usually made for specimen extraction [9, 11, 14, 15, 17]. Given previous studies reporting a comparable wound complication rate between left lower transverse incisions and vertical incisions [14, 15], the iliac fossa area is considered an appropriate specimen extraction site for laparoscopic radical resection. Although the right lower transverse incision is reported to be an extraction site in single-incision laparoscopic left-sided colorectal resection [18, 19], and in radical proctectomy when the specimen extraction site is also used as the stoma site [20], few studies have addressed the usefulness of the right lower transverse area as an extraction site in patients undergoing laparoscopic surgery for left-sided colorectal cancer.

Therefore, this study was to evaluate the perioperative outcomes of a right lower transverse incision for laparoscopic surgery for left-sided colorectal cancer, compared with a vertical transumbilical incision.

## Methods

Two hundred twelve consecutive patients with rectosigmoid and upper rectal cancer underwent laparoscopic resection between July 2010 and December 2014 and were registered prospectively into the database. Upper rectal cancer was defined as a tumor located above the anterior peritoneal reflection based on operative findings. Of the 212 patients, 29 were excluded for the following reasons: no anastomosis (*n* = 14), surgery for perforation or obstruction (*n* = 13), and laparoscopic resection with specimen extraction through the anus (*n* = 2). The data of the remaining 183 patients were analyzed.

**Fig. 1:**
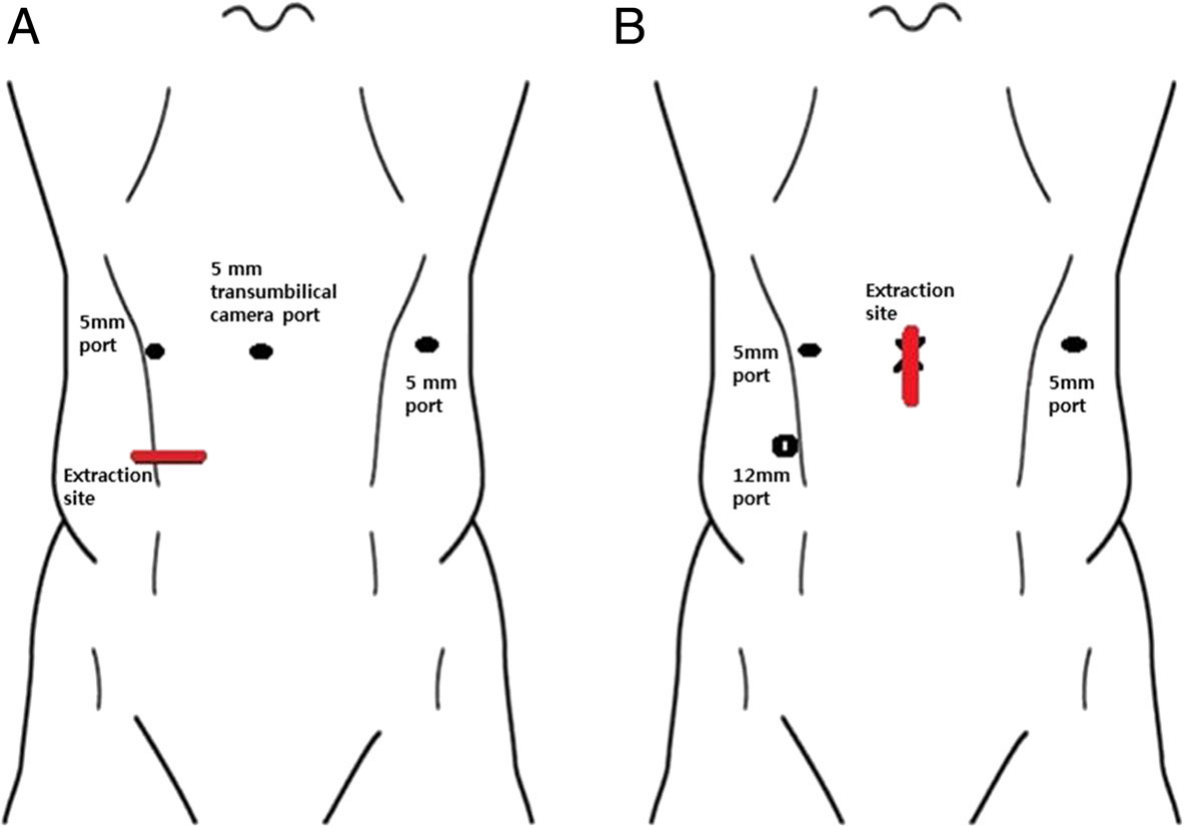
**a** Port placement for patients who underwent laparoscopic resection using a right lower transverse incision. **b** Port placement for patients who underwent laparoscopic resection using a vertical transumbilical incision

All operations were performed by one colorectal surgeon with considerable experience in laparoscopic colorectal surgery (more than 1000 cases). Initially, a mini-laparotomy incision of about 4 cm was made at the right lower quadrant or vertical transumbilicus similar to the technique of single-incision laparoscopic colectomy. The incision length was decided according to tumor size. A right lower transverse incision was preferred if the tumors were located distally or a protective stoma was expected. After deployment of a wound protector (Alexis® wound retractor, Applied Medical, Rancho Santa Margarita, CA, USA) into the incision site, a surgical glove was installed. The surgical glove port was constructed by fixing two trocars (one 12-mm trocar and one 5-mm trocar) to the resected finger tip of the glove. A pneumoperitoneum was then established at a pressure of 10 to 12 mmHg. In all patients, a 0° 5-mm rigid laparoscope was used. A laparoscope was introduced through the surgical glove port, and additional trocars were inserted under laparoscopic vision. In patients who underwent laparoscopic surgery using a right lower transverse incision, a transumbilical 5-mm trocar was inserted for the laparoscopic camera, one 5-mm trocar was inserted for the operator’s use in the right midabdomen in the midclavicular line, and another 5-mm trocar was inserted for the assistant’s use in the left midabdomen lateral to the rectus muscle. In patients who underwent laparoscopic surgery using a transumbilical incision, one 12-mm trocar was inserted in the right lower abdomen and two 5-mm trocars were inserted, one in the right midabdomen in the midclavicular line and one in the left midabdomen lateral to the rectus muscle (Fig. 1). With the patients positioned in a slight Trendelenburg positioning with a right lateral tilt, laparoscopic electrocautery was used to score the peritoneum at the sacral promontory level, immediately dorsal to the pedicle of the inferior mesenteric artery. Next, the inferior mesenteric artery was ligated close to its origin while avoiding injury to the superior hypogastric nerve plexus. The inferior mesenteric vein was divided as close to the inferior border of the pancreas as possible. The dissection was discontinued when the left colon and retroperitoneum were separated, and all parts of colonic mesentery were transected intracorporeally using a vessel-sealing device. In patients with upper rectal cancer, a partial mesorectal excision was accomplished and the rectum was transected using one or two laparoscopic linear staplers. Following all laparoscopic procedures, the specimen was extracted through the initial right lower transverse or vertical transumbilical incision. Some patients underwent performance of a long right lower transverse incision to extract bulky specimens and create a stoma. In such cases, the appropriate stoma size was ensured by suturing this long abdominal incision. After extracorporeal transection of the proximal colon, intracorporeal anastomosis was usually performed with a circular stapler. Following this, four to six intracorporeal reinforcement sutures were performed in nearly all patients.

The 183 patients that underwent laparoscopic resection for rectosigmoid or upper rectal cancer were divided into two groups according to the specimen extraction site: right lower transverse incision group (RLT group, *n* = 61) or vertical transumbilical incision group (VTU group, *n* = 122). The clinical parameters analyzed included patient characteristics and perioperative outcomes, including operative time, intraoperative blood loss, numbers of lymph nodes examined, time to first flatus, postoperative pain scores, and postoperative length of hospital stay. Maximum pain was assessed on days 1 to 7 postoperatively using a visual analog scale, and the scores obtained were used to assess postoperative pain severity. The operative time was defined as the duration from performance of the first incision to completion of wound closure. The intraoperative blood loss volume was determined by subtracting the volume of instilled fluids from the volume of aspirated fluids. Diverting stomas were constructed at the surgeon’s discretion. Anastomotic leakage was considered present when a patient had fluid collection in the abdominopelvic cavity seen on imaging or by clinical symptoms (fever, leukocytosis, and purulent or fecal discharge from the drain) requiring operative or radiological intervention. Incisional hernia was defined as a palpable defect at the laparoscopic extraction or stoma site found on follow-up clinical examination. For patients that underwent diverting stoma, the development of an incisional hernia at the extraction site was evaluated after stoma closure. Parastomal hernia was defined as a palpable defect or bulge adjacent to a stoma. Wound infection was considered to be present when purulent discharge from a wound was observed or culture of the discharge was positive. Mortality was defined as death either during hospitalization or ≤30 days postoperatively. The ethics committee of Inje University Haeundae Paik Hospital approved the study protocol, and written informed consent was obtained from all patients before surgery.

### Statistical analysis

Propensity score matching was performed to compensate for differences in the clinicopathological characteristics between the RLT (*n* = 61) and VTU (*n* = 122) groups. The 10 covariables entered into the propensity model were age, gender, body mass index (BMI), American Society of Anesthesiologists (ASA) score, previous laparotomy, incisional length, tumor location, tumor size, differentiation of tumor, and TNM stage. Propensity scores were calculated using a logistic regression method. A 1:1 match between the RLT and VTU groups was performed using the nearest neighbor matching technique. After matching, baseline characteristics and perioperative outcomes were compared using chi-square test or Fisher’s exact test and Wilcoxon’s rank sum test. A *p* value of <0.05 was considered statistically significant. All analyses were performed using SAS software ver. 9.3 (SAS Institute, Cary, NC, USA).

## Results

Overall, 183 patients were included in the study, of whom 61 patients (33.3 %) underwent laparoscopic colectomy with specimen extraction through a right lower transverse incision. The median age of the 183 study subjects was 65.0 years (range 31 to 92 years), median body mass index was 23.7 kg/m^2^ (range 15.2 to 34.9 kg/m^2^), median tumor size was 4.0 cm (range 2.5 to 9.0 cm), and median follow-up duration was 31 months (range 11 to 63 months). Before propensity score matching, the patients that received a right lower transverse incision or a vertical transumbilical incision were significantly different with respect to tumor location, tumor size, and the proportion of patients older than 70 years. However, after matching, no significant differences were found between patient characteristics in the RLT and VTU groups (Table 1).

**Table 1:** Clinicopathological characteristics of patients before and after propensity score matching

Variables		Total cohort			Matched cohort		
		RLT group	VTU group	*p* value	RLT group	VTU group	*p* value
		(*n =* 61)	(*n =* 122)		(*n =* 57)	(*n =* 57)	
Age (years)	≤70	37 (60.7)	95 (77.9)	0.014^a^	37 (64.9)	41 (71.9)	0.420^a^
	>70	24 (39.3)	27 (22.1)		20 (35.1)	16 (28.1)	
Gender	Male	40 (65.6)	75 (61.5)	0.589^a^	39 (68.4)	37 (64.9)	0.691^a^
	Female	21 (34.4)	47 (38.5)		18 (31.6)	20 (35.1)	
ASA grade	I–II	50 (82.0)	107 (87.7)	0.295^a^	47 (82.5)	45 (79.0)	0.635^a^
	III	11 (18.0)	15 (12.3)		10 (17.5)	12 (21.0)	
Previous history of laparotomy		16 (26.2)	20 (16.4)	0.115^a^	14 (24.6)	12 (21.1)	0.655^a^
Tumor location	RSC	21 (34.4)	69 (56.6)	0.005^a^	21 (36.8)	23 (40.4)	0.700^a^
	UR	40 (65.6)	53 (43.4)		36 (63.2)	34 (59.7)	
BMI (kg/m^2^, range)		23.4	23.9	0.243^b^	23.4	24.0	0.903^b^
		(18.3–34.9)	(15.2–33.4)		(18.3–30.1)	(17.3–30.8)	
Incision length (cm, range)		4.0 (3.0–6.0)	4.0 (2.5–9.0)	0.143^b^	4.0 (3.0–6.0)	4.0 (2.5–9.0)	0.505^b^
Tumor size (cm, range)		5.1 (0.2–9.5)	3.7 (0.5–13.0)	0.005^b^	5.0 (0.2–9.5)	5.0 (0.5–13.0)	0.803^b^
Histology	WD/MD	58 (95.1)	118 (96.7)	0.688^c^	55 (96.5)	55 (96.5)	1.000^c^
	PD	3 (4.9)	4 (3.3)		2 (3.5)	2 (3.5)	
Stage	I	12 (19.7)	37 (30.3)	0.387^c^	12 (21.1)	12 (21.1)	0.886^c^
	II	16 (26.2)	31 (25.4)		16 (28.1)	13 (22.8)	
	III	31 (50.8)	52 (42.7)		28 (49.1)	30 (52.6)	
	IV	2 (3.3)	2 (1.6)		1 (1.8)	2 (3.5)	

Values are expressed as median or as numbers and percentages; numbers in parenthesis are percentages unless otherwise denoted

*RLT* right lower transverse incision, *VTU* vertical transumbilical incision, *ASA* American Society of Anesthesiologists, *RSC* rectosigmoid colon, *UR* upper rectum, *BMI* body mass index, *WD* well-differentiated tumor, *MD* moderately differentiated tumor, *PD* poorly differentiated tumor

^a^ *p* values were calculated using chi-square test

^b^ *p* values were calculated using Wilcoxon’s rank sum test

^c^ *p* values were calculated using Fisher’s exact test

Table 2 shows the perioperative outcomes in the matched cohorts. Median numbers of harvested lymph nodes were 24.0 in the RLT group and 22.0 in the VTU group (*p* = 0.905), and median distal margin lengths, operative times, and intraoperative blood losses were similar in the two groups (*p* = 0.930, *p =* 0.650, *p =* 0.520, respectively). Conversion to open surgery was not required in either group, and proportions of patients with extraperitoneal anastomosis were similar (52.6 vs 43.8 %, *p =* 0.454). Although percentages requiring diverting stoma were not significantly different (26.3 vs 14.0 %, *p =* 0.161), the percentage requiring an additional incision for diverting stoma was higher in the VTU group (14.0 vs 0.0 %, *p =* 0.003).

**Table 2:** Comparison of perioperative outcomes

Variables	RLT group	VTU group	*p* value
	(*n =* 57)	(*n =* 57)	
Numbers of harvested LN (range)	24.0 (4–63)	22 (1–75)	0.905^a^
Distal resection margin (cm, range)	5.0 (0.4–8.0)	4.8 (0.2–8.5)	0.930^a^
Proximal resection margin (cm, range)	11.0 (3.7–64.5)	12.0 (1.5–30.5)	0.565^a^
Operative time (min, range)	255.0 (150.0–535.0)	250.0 (160.0–450.0)	0.650^a^
Estimated blood loss (mL, range)	100.0 (10.0–850.0)	100.0 (50.0–1000)	0.520^a^
Splenic flexure mobilization (%)	15 (26.3)	22 (38.6)	0.230^b^
Extraperitoneal anastomosis (%)	30 (52.6)	25 (43.8)	0.454^b^
Diverting stoma creation (%)	15 (26.3)	8 (14.0)	0.161^b^
Conversion to open surgery (%)	0 (0.0)	0 (0.0)	1.000^c^
Additional incision for diverting stoma (%)	0 (0.0)	8 (14.0)	0.003^c^

Values are expressed as median numbers

*RLT* right lower transverse incision, *VTU* vertical transumbilical incision

^a^ *p* values were calculated using Wilcoxon’s rank sum test

^b^ *p* values were calculated using chi-square test

^c^ *p* values were calculated using Fisher’s exact test

Postoperative courses after matching are shown in Table 3. No significant intergroup differences were observed in the time to first flatus, time to resume soft diet, or length of hospital stay. Maximal pain scores during the early postoperative period (1 to 7 days) were similar in the two groups (*p =* 0.244).

**Table 3:** Postoperative courses

Variables	RLT group	VTU group	*p* value^a^
	(*n =* 57)	(*n =* 57)	
Time to first flatus (days, range)	3.0 (1.0–6.0)	3.0 (1.0–14.0)	0.657
Time to resumed soft diet (days, range)	6.0 (2.0–11.0)	7.0 (3.0–16.0)	0.150
Postoperative length of hospital stay (days, range)	14.0 (9.0–36.0)	14.0 (8.0–56.0)	0.411
Postoperative pain score (VAS, range)^b^	6.0 (1.0–9.0)	6.0 (1.0–10.0)	0.244

*RLT* right lower transverse incision, *VTU* vertical transumbilical incision, *VAS* visual analog scale

^a^ *p* values were calculated using Wilcoxon’s rank sum test

^b^Maximum VAS scores on days 1 to 7 after surgery were used to assess postoperative pain severity

In addition, overall surgical complication rates in the RLT and VTU groups were similar (24.6 and 19.3 %, respectively, *p =* 0.497). Incisional hernia rates (3.5 vs 1.8 %, *p =* 0.558) and surgical site infection rates (5.3 vs 3.5 %, *p =* 0.647) were comparable. Parastomal hernias in the cases of diverting stoma developed in six of 15 patients in the RLT group and one of eight patients in the VTU group (*p =* 0.172). Three anastomotic leakages occurred in the VTU group and none in the RLT group, which was not statistically significant. Of the three patients in the VTU group that developed anastomotic leakage, laparoscopic stoma creation and irrigation was performed in one and the other two were managed conservatively (Table 4).

**Table 4:** Surgical complications

	RLT group	VTU group	*p* value
	(*n =* 57)	(*n* = 57)	
Overall	14 (24.6)	11 (19.3)	0.497^a^
Chylous ascites	2 (3.5)	2 (3.5)	1.000^b^
EPSBO	1 (1.8)	1 (1.8)	1.000^b^
Anastomotic leakage	0 (0.0)	3 (5.3)	0.079^b^
Surgical site infection	3 (5.3)	2 (3.5)	0.647^b^
Incisional hernia at extraction site	2 (3.5)	1 (1.8)	0.558^b^
Parastomal hernia	6/15 (40.0)	1/8 (12.5)	0.172^b^
Reoperation for surgical complications	0 (0.0)	1 (1.8)	0.315^b^
Mortality	0 (0.0)	0 (0.0)	1.000^b^

Values in parenthesis are percentages

*RLT* right lower transverse incision, *VTU* vertical transumbilical incision, *EPSBO* early postoperative small bowel obstruction

^a^ *p* value was calculated using chi-square test

^b^ *p* values were calculated using Fisher’s exact test

## Discussion

The ideal location for specimen extraction following laparoscopic colorectal surgery remains controversial. This case-matched study shows that a right lower transverse incision produces results similar to those of a vertical transumbilical incision in patients that undergo laparoscopic resection for rectosigmoid or upper rectal cancer in terms of perioperative outcomes, postoperative course, and surgical morbidity. However, the rate of additional incision for requiring diverting stoma was significantly lower in the RLT group. Therefore, given the increasing trend toward minimizing surgical trauma during laparoscopic surgery for colorectal cancer [21, 22], the potential advantage offered by right lower transverse extraction over transumbilical extraction is that it may reduce the need for an additional incision.

Previous studies have compared the incidences of incisional hernia after transverse or vertical incisions in laparoscopic colorectal surgery and demonstrated that a transverse extraction site is associated with a significantly lower risk of incisional hernia development [9, 10]. However, in the present study, the rates of incisional hernia were similar in the RLT and VTU groups (5.3 vs 3.5 %, *p =* 0.558). This finding compares well with those of previous studies, which showed no significant difference in incisional hernia rates between the transverse and vertical incisions in laparoscopic colectomy for left-sided colorectal cancer [14, 15]. Because BMIs and tumor locations in these previous studies were similar to those in the present study, it is therefore postulated that the transverse and vertical incisions used for laparoscopic extraction sites could be performed in left-sided colorectal cancer patients with a BMI of <25 kg/m^2^.

Previous studies [14, 15] that compared short-term outcomes following laparoscopic resection in left-sided colorectal cancer with left transverse and vertical mini-laparotomy groups indicated a weak association between extraction site and infectious postoperative complications. The present study showed similar results for rates of anastomotic leakage and surgical site infection when comparing the RLT group and the VTU group. Furthermore, no harmful effects of a right lower incision were demonstrated over a vertical incision in terms of infection-associated morbidities. These consistent infectious morbidity rates suggest that the specimen extraction and extracorporeal fashioning of anastomoses can be safely performed through a right lower extraction site. The rates of infectious complications after a right lower transverse incision for laparoscopic left-sided colorectal resection were 0.0 % for anastomotic leakages and 5.3 % for surgical site infections in this study, whereas published values for left lower transverse incisions in left-sided colorectal cancer range from 4 to 5.5 % for anastomotic leakages and 10 to 12.7 % for surgical site infections [14, 15].

In studies that focused on specimen extraction sites for laparoscopic left-sided colorectal resection [11, 14, 15], the authors preferred vertical or low transverse incisions (either a left lower transverse or Pfannenstiel incision). The reasons given for a left lower transverse incision were not clear, but it seemed to be related to potential difficulty in mobilizing the splenic flexure during laparoscopic left-sided colorectal resection. Given the similar percentages of patients that required splenic flexure mobilization in the two groups of this study, it is believed that the use of a right lower transverse incision for laparoscopic left-sided radical colorectal resection has no detrimental impact on splenic flexure mobilization. Karakayali et al. [20] suggested a right lower transverse incision for laparoscopic radical proctectomy had advantages over a Pfannenstiel incision with respect to additional mini-laparotomy, cosmesis, and postoperative course. These suggestions and findings in the present study support the opinion that a right lower transverse incision can be used as an alternative to other transverse incisions such as Pfannenstiel and left lower transverse incisions.

The incidence of parastomal hernia during stoma closure in the RLT group was 40 %, which is high compared with the 3–19 % values reported in previous series that studied the feasibility and safety of right and left lower transverse incisions for specimen extraction and simultaneous stoma sites during laparoscopic radical proctectomy [20, 23]. Although several factors affect the parastomal hernia rate, the rectus muscle cutting technique used in the present study may have been responsible for the high rate observed. In fact, Navaratnam et al. [24] postulated that the muscle cutting technique used to create a transverse extraction site may be associated with a higher probability of incisional hernia. However, because parastomal hernia can be repaired during stoma closure surgery, its development may be considered a minor postoperative event.

This study has some limitations. First, although propensity score matching was performed to control for confounders, residual selection bias is likely. Further, the relatively small number of patients enrolled could have prevented detecting significant differences between morbidity outcomes. Second, because the study did not compare short-term outcomes of right lower transverse and other types of low transverse incisions in laparoscopic left-sided colorectal resection, further comparative study is required to determine the optimal site for a low transverse incision.

## Conclusions

A right lower transverse incision appeared to be as effective as a vertical transumbilical incision in terms of short-term outcomes after laparoscopic surgery for left-sided colorectal cancer and may be considered as a preferred extraction site because it lowered the risk of additional mini-laparotomy for diverting stoma.

## Abbreviations

RLT: Right lower transverse incision
VTU: Vertical transumbilical incision
